# A systematic comparison of sugar content in low-fat vs regular versions of food

**DOI:** 10.1038/nutd.2015.43

**Published:** 2016-01-25

**Authors:** P K Nguyen, S Lin, P Heidenreich

**Affiliations:** 1 Department of Medicine, Division of Cardiovascular Medicine, Stanford University, Stanford, CA, USA; 2 Veterans Affairs Palo Alto Medical Center, Palo Alto, CA, USA

## Abstract

Obesity remains a significant public health concern. One of the primary messages from providers and health-care organizations is to eat healthier foods with lower fat. Many in the lay press, however, have suggested that lower fat versions of foods contain more sugar. To our knowledge, a systematic comparison of the sugar content in food with lower fat alternatives has not been performed. In this study, we compared fat free, low fat and regular versions of the same foods using data collected from the USDA National Nutrient Database. We found that the amount of sugar is higher in the low fat (that is, reduced calorie, light, low fat) and non-fat than ‘regular' versions of tested items (Friedman *P*=0.00001, Wilcoxon *P*=0.0002 for low fat vs regular food and *P*=0.0003 for non-fat vs regular food). Our data support the general belief that food that is lower in fat may contain more sugar.

## Background

Obesity remains a significant public health concern in the United States (US). Conventional thinking suggests that obesity is caused by an imbalance between calories consumed and calories expended; thus, any excess of calories will result in obesity. The solution is simple in theory: eat less and exercise more. Despite numerous recommendations on how to achieve this goal, a third of adults and 17% of children are obese.^1^ Perhaps it is not only how much we are eating, but also what we are eating that may be encouraging our bodies to store fat.

The impetus for creating low-fat foods can be traced to the McGovern Committee, which issued a report in 1977 recommending that Americans eat less fat and more complex carbohydrates to prevent diabetes, heart disease and stroke. Over the subsequent decades, several health advocacy groups have echoed these same recommendations, giving rise to America's current aversion to fatty foods. However, the food industry may have replaced fat with sugar, which may be more obesogenic even if the calories per portion are less.^2, 3^ The purpose of this study is to determine whether these ‘healthier' versions of common foods have more sugar than their ‘regular' counterparts.

## Materials and methods

We evaluated the nutrient value of the list of foods recommended by the National Heart Lung and Blood Institute on a website entitled ‘Low-calorie, lower fat alternative foods' (http://www.nhlbi.nih.gov/health/educational/lose_wt/eat/shop_lcal_fat.htm). The site lists high-calorie/high-fat foods that have low-calorie, non-fat alternatives. We compared the nutritional content of the same foods listed on the USDA National Nutrient Database for Standard Reference released in 2004 (SR17)^4^ and 2014 (SR27).^5^ SR17 was chosen as the initial data set for comparison because it is the earliest version with the most complete nutrient information. This database lists the nutritional content of over 8000 generic and brand name food products and is the major source of food composition data in the US. The database includes information on the mean nutrient values per 100 g of the edible portion of food including the amount of protein, fat and carbohydrates. Continuous variables were represented as medians with interquartile range. Differences among and between food groups were analyzed using the Friedman test, followed by *post hoc* Wilcoxon signed-rank test, respectively. Statistical analysis was performed using STATA, version 12.1 (STATA, College Station, TX, USA). Significance level was set at *P*<0.05.

## Results

**Figure 1:**
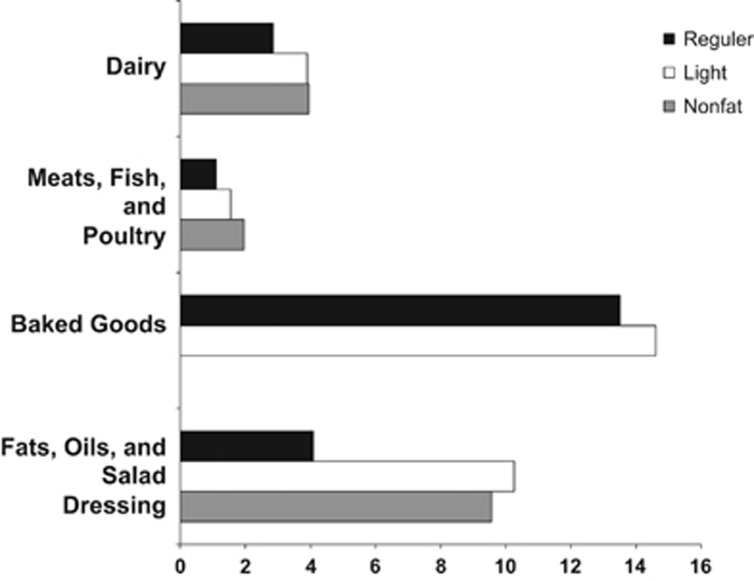
Bar graph showing the median sugar content (g) of selected food items grouped by major food categories. Data taken from USDA National Nutrient Database released in 2014. Dairy: regular vs low calorie (*P*=0.011) vs non fat (*P*=0.036); meat, fish and poultry: regular vs low calorie (*P*=0.080) vs non fat (*P*=0.043); baked goods: regular vs low calorie (*P*=0.0180); fats, oils and salad dressings: regular vs low calorie (*P*=0.091) vs non fat (*P*=0.0180).

On the basis of the information collected by the USDA, we found that the amount of sugar is higher in the low-fat (that is, reduced calorie, light, low fat) and non-fat than regular versions of tested items (Friedman *P*=0.00001, Wilcoxon *P*=0.0002 for low-fat vs regular food and *P*=0.0003 for non-fat vs regular food). Subgroup analysis revealed that sugar content was higher in lower calorie versions of the following food categories: (i) dairy products, (ii) baked goods, (iii) meats, fish and poultry, and (iv) fats, oils and salad dressings (Figure 1 and Table 1). Results did not significantly differ between 2004 and 2014 (data not shown).

## Discussion

Findings from this study suggest that consuming foods lower in fat have higher sugar content despite having lower calories. Although the increase in added sugar per serving appears to be small, the cumulative effect of consuming ‘empty calories' over several years could have important health consequences.

Consuming excess sugar even in small amounts (⩾10% of total calories) has been shown to be harmful, leading to weight gain, diabetes and cardiovascular disease.^2, 3^ The major sources of added sugar in the diet include the obvious culprits like sugar-sweetened beverages, desserts, fruit drinks and candy.^2, 3^ Ironically, individuals who believe they are choosing healthier versions of their favorite foods are trading fat for less healthy sugar. Although exchanging sugar for fat alone may not increase rates of obesity as shown in a recent systematic review,^6^ eating food high in sugar may promote consumption of excess calories by inducing leptin resistance and increasing the risk of obesity.^7^

## Figures and Tables

**Table 1:** List of main nutrient values (per 100 g) in common regular, low-fat and non-fat foods in the USDA National Nutrient Database for Standard Reference in 2014

*Items*	*Serving size (g)*	*Protein (g)*			*Fat (g)*			*Carbohydrates (g)*			*Sugar (g)*			*Energy (kcal)*		
		*Regular*	*Light*	*Non fat*	*Regular*	*Light*	*Non fat*	*Regular*	*Light*	*Non fat*	*Regular*	*Light*	*Non fFat*	*Regular*	*Light*	*Non fat*
*Dairy*																
Milk	405 (1 cup)	3.15	3.37	3.37	3.27	0.97	0.08	4.80	4.99	4.96	5.05	5.20	5.09	61	42	34
Yogurt, plain	245 (1 cup)	3.47	5.25	5.73	3.25	1.55	0.18	4.66	7.04	7.68	4.66	7.04	7.68	61	63	56
Kraft breakstone's sour cream	15 (1 tbsp)	0	4.50	4.70	16.13	12.00	1.30	3.23	6.50	15.10	3.23	6.40	7.20	158	152	91
Cottage cheese	113 (4 oz)	10.90	10.90	10.34	4.20	1.00	0.29	3.00	3.00	6.66	0.37	3.00	1.85	95	151	NA
Kraft cheez whiz	33 (2 tbsp)	12.00	16.30	NA	21.00	9.50	NA	9.20	16.20	NA	6.70	8.20	NA	276	215	NA
Cream cheese	20 (1 tbsp)	5.93	7.85	15.69	34.24	15.28	1.00	4.07	8.13	7.66	3.21	5.82	5.48	342	201	105
Mozzarella cheese	28 (1 slice)	22.17	24.26	31.7	22.35	15.92	0.00	2.19	2.77	3.5	1.03	1.13	1.48	300	254	141
Cheddar cheese	28 (1 slice)	24.04	27.35	32.4	33.82	20.41	0.00	1.33	4.06	7.14	0.28	0.26	0	406	309	157
Swiss cheese	28 (1 slice)	24.73	25.50	28.40	25.01	5.10	0.00	2.10	4.30	3.40	1.23	1.35	1.33	380	179	127

*Meat, fish and poultry*																
Oscar Mayer (bologna, beef)	28 (1 slice)	11.05	11.75	12.6	29.1	14.50	0.6	2.45	5.6	6	1.4	2.3	2.2	1322	835	331
Oscar Mayer (beef franks)	28 (1 slice)	11.35	10.7	13.2	30.26	14.90	0.5	2.78	4.1	5.1	1.6	2.1	3.8	329	193	78
Oscar Mayer (smoked ham)	28 (1 slice)	16.60	16.30	14.60	10.46	3.52	0.70	0.10	1.83	1.90	0.10	1.00	1.10	99	104	72
Louis Rich turkey breast (oven roasted)	28 (1 slice)	17.10	19.10	15.00	2.00	0.70	0.70	3.25	1.90	4.50	0.80	0.40	1.70	99	90	84

*Baked goods including snacks and sweets*																
Cheese crackers	39 (4 crackers)	7	7.8	NA	24.9	17.5	NA	60	68	NA	13.50	14.6	NA	494	461	NA
Brownies	56 (1 brownie)	4.8	2.77	NA	16.3	9.68	NA	63.90	61.58	NA	36.61	38.73	NA	1695	1441	NA
Chocolate cookies with crème filling	12 (1 cookie)	5.21	2.94	NA	19.14	13.24	NA	71	76.17	NA	40.67	41.18	NA	464	436	NA
Keebler town crackers	16 (5 crackers)	5.10	6.60	NA	29.60	11.30	NA	59.80	77.10	NA	6.50	8.40	NA	526	431	NA
Keebler, vienna fingers with crème filling	15.5 (1 cookie)	4.5	4.5	NA	20.1	15	NA	73.2	78.1	NA	33.6	37.2	NA	492	465	NA
Sunshine cheez-it	5.56 (1 cracker)	11.4	13.3	NA	26.3	14.5	NA	57.4	67	NA	0.5	0.8	NA	507	449	NA
Kellogg's eggo waffles, homestyle	35 (1 waffle)	6.60	6.30	NA	10.55	3.50	NA	39.80	44.90	NA	2.70	4.50	NA	278	229	NA

*Fats, oils and salad dressing*																
Mayonnaise dressing, no cholesterol	13.8 (1 tbsp)	0	0.9	0.20	77.8	33.3	15.50	0.3	6.7	2.70	0.3	4.2	10.30	680	4.2	84
Ranch salad dressing	15 (1 tbsp)	1.32	1.25	1.92	44.54	12.42	26.51	5.74	21.33	3	4.69	3.77	5.35	1763	3.77	497
Thousand island salad dressing	16 (1 tbsp)	1.09	0.83	0.55	35.06	11.32	1.45	14.64	24.06	29.27	15.18	17.31	16.83	379	17.31	132
French dressing	16 (1 tbsp)	0.77	0.58	0.20	44.81	11.52	0.27	15.58	31.22	32.14	15.95	16.86	16.45	457	222	132
Blue cheese salad dressing	15 (1 tbsp)	1.37	5.1	1.52	51.1	7.2	1.01	4.7	13.2	25.06	3.48	2.82	7.44	NA	NA	NA
Caesar salad dressing	14.7 (1 tbsp)	2.17	0.3	1.47	57.85	4.4	0.23	3.3	18.6	30.73	2.81	16.32	8.82	542	16.32	134

Abbreviations: NA, not applicable; tbsp, tablespoon.
